# Bentall procedure for giant unruptured right sinus of Valsalva aneurysm treated

**DOI:** 10.1093/jscr/rjag038

**Published:** 2026-01-30

**Authors:** Jeonga Lee, Ryohei Ushioda, Hidenobu Akamatsu, Tasuku Kawarabayashi, Akito Inoue, Kaname Shimizu, Kentaro Shirakura, Yuki Setogawa, Ryo Okubo, Hiroyuki Miyamoto, Aina Hirofuji, Daisuke Takeyoshi, Shogo Takahashi, Shingo Kunioka, Hiroyuki Kamiya

**Affiliations:** Department of Cardiac Surgery, Asahikawa Medical University, MMidorigaoka 1-1-1, Asahikawa, Hokkaido 078-8510, Japan; Department of Cardiac Surgery, Asahikawa Medical University, MMidorigaoka 1-1-1, Asahikawa, Hokkaido 078-8510, Japan; Department of Cardiac Surgery, Asahikawa Medical University, MMidorigaoka 1-1-1, Asahikawa, Hokkaido 078-8510, Japan; Department of Cardiac Surgery, Asahikawa Medical University, MMidorigaoka 1-1-1, Asahikawa, Hokkaido 078-8510, Japan; Department of Cardiac Surgery, Asahikawa Medical University, MMidorigaoka 1-1-1, Asahikawa, Hokkaido 078-8510, Japan; Department of Cardiac Surgery, Asahikawa Medical University, MMidorigaoka 1-1-1, Asahikawa, Hokkaido 078-8510, Japan; Department of Cardiac Surgery, Asahikawa Medical University, MMidorigaoka 1-1-1, Asahikawa, Hokkaido 078-8510, Japan; Department of Cardiac Surgery, Asahikawa Medical University, MMidorigaoka 1-1-1, Asahikawa, Hokkaido 078-8510, Japan; Department of Cardiac Surgery, Asahikawa Medical University, MMidorigaoka 1-1-1, Asahikawa, Hokkaido 078-8510, Japan; Department of Cardiac Surgery, Asahikawa Medical University, MMidorigaoka 1-1-1, Asahikawa, Hokkaido 078-8510, Japan; Department of Cardiac Surgery, Asahikawa Medical University, MMidorigaoka 1-1-1, Asahikawa, Hokkaido 078-8510, Japan; Department of Cardiac Surgery, Asahikawa Medical University, MMidorigaoka 1-1-1, Asahikawa, Hokkaido 078-8510, Japan; Department of Cardiac Surgery, Asahikawa Medical University, MMidorigaoka 1-1-1, Asahikawa, Hokkaido 078-8510, Japan; Department of Cardiac Surgery, Asahikawa Medical University, MMidorigaoka 1-1-1, Asahikawa, Hokkaido 078-8510, Japan; Department of Cardiac Surgery, Asahikawa Medical University, MMidorigaoka 1-1-1, Asahikawa, Hokkaido 078-8510, Japan

**Keywords:** sinus of Valsalva aneurysm, unruptured aneurysm, Bentall procedure

## Abstract

A sinus of Valsalva aneurysm (SVA) is a rare cardiac anomaly that may remain silent until rupture, often leading to acute heart failure. Surgical intervention is advised for unruptured SVAs when large, progressive, or associated with aortic regurgitation (AR). We report a 72-year-old woman with a 25-mm right coronary SVA and severe symptomatic AR. Transthoracic echocardiography confirmed severe AR with preserved ventricular function. A Bentall procedure using a bioprosthetic valve and Valsalva graft was performed, with coronary button reimplantation with the Carrel patch technique. The postoperative course was uneventful, and the patient remained free of recurrence at 2-year follow-up. This case highlights the Bentall procedure as a durable option for unruptured SVA with root distortion and valve involvement, offering reliable prevention of late AR and reintervention.

## Introduction

A sinus of Valsalva aneurysm (SVA) is a rare cardiac anomaly caused by enlargement of the aortic root between the aortic valve annulus and the sinus tubular junction [1]. Most SVAs remain asymptomatic until rupture, at which point they often present with acute heart failure or continuous murmurs due to left-to-right shunting [2]. However, in unruptured cases, surgical intervention is considered when the aneurysm is large, shows progressive enlargement, or is associated with aortic regurgitation (AR) or coronary artery involvement.

Here, we report a case of a giant unruptured right coronary SVA associated with severe AR, successfully treated with the Bentall procedure.

## Case presentation

**Figure 1:**
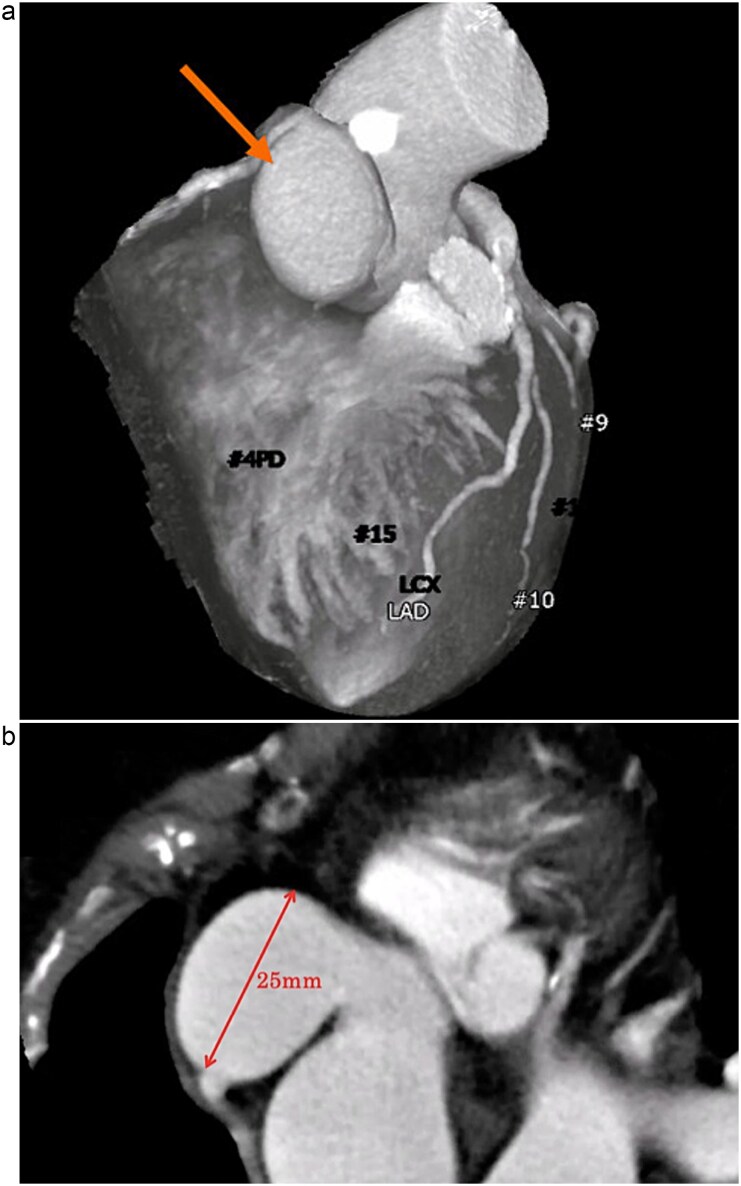
(a) Contrast-enhanced CT demonstrating a saccular aneurysm arising from the right coronary sinus of Valsalva (arrow), without evidence of significant coronary artery disease. (b) Axial contrast-enhanced CT image showing the saccular aneurysm, with a maximal diameter of 25 mm.

**Figure 2:**
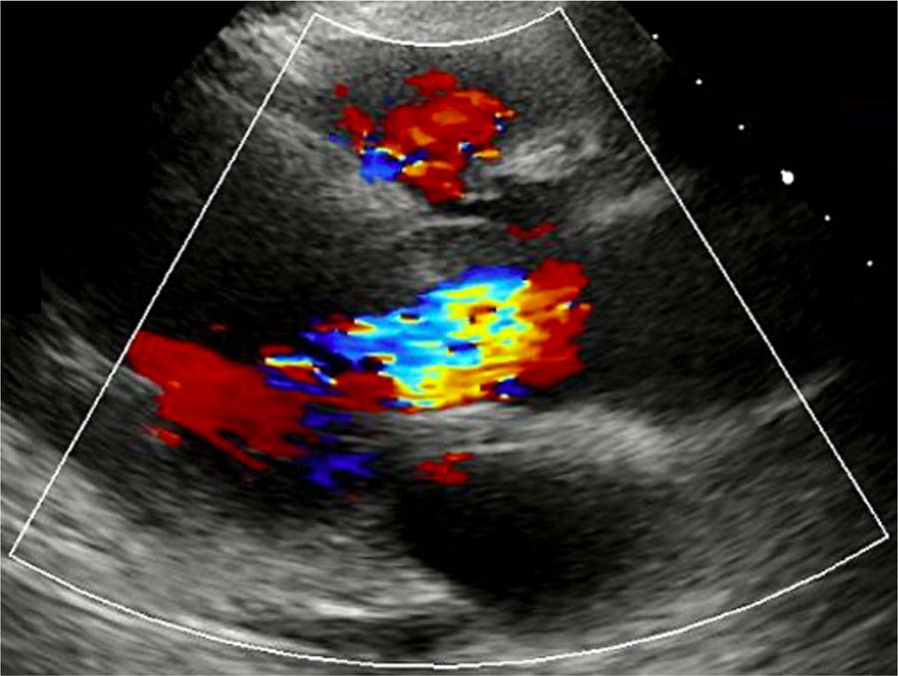
TTE revealed severe AR.

A 72-year-old woman with hypertension and dyslipidemia presented with progressive dyspnea. There was no family history of congenital heart disease. Electrocardiography showed sinus rhythm at 67 bpm and complete right bundle branch block. Contrast-enhanced computed tomography (CT) revealed a 25-mm saccular aneurysmal dilation arising from the right coronary sinus of Valsalva, without significant coronary artery stenosis (Fig. 1a and b). Transthoracic echocardiography (TTE) revealed severe AR (a regurgitant volume of 91 ml, an effective regurgitant orifice area of 0.54 cm^2^, and a maximum regurgitant velocity of 3.4 m/s). Left ventricular systolic function was preserved, with a left ventricular end-diastolic/systolic diameter of 55/38 mm, and no ventricular septal defect was detected (Fig. 2). Although unruptured, the aneurysm was giant and associated with severe, symptomatic AR; therefore, surgical intervention was indicated. Given an acceptable operative risk (preoperative European System for Cardiac Operative Risk Evaluation II, 3.48%), a modified Bentall procedure was performed.

Cardiopulmonary bypass was established via arterial cannulation of the ascending aorta and venous cannulation of the right atrium. The aneurysmal wall of right SVA was markedly dilated and extremely thinned. Inspection of the aortic valve revealed diffuse leaflet thickening without commissural distortion, cusp prolapse, or global annular dilatation; therefore, a Bentall procedure was performed as planned. It was performed using a 23 mm INSPIRIS valve (Edwards Lifesciences Corporation, Irvine, CA, USA) and a 26 mm Gelweave Valsalva graft (Vascutek Terumo, Inchinnan, UK). Coronary buttons were fashioned and reimplanted into the composite graft using the Carrel patch technique. Histopathological examination of the resected aneurysmal wall demonstrated medial thinning with loss of smooth muscle cells and elastic fiber fragmentation, without evidence of active inflammation, supporting degenerative etiology.

No residual AR was noted by TTE postoperatively. The postoperative course was uneventful, and the patient was discharged on postoperative day 15. She has remained free of aneurysm and AR recurrence at the 2-year follow-up.

## Discussion

SVA is a rare cardiac anomaly, accounting for ~0.1%–3.5% of all congenital heart defects and 0.14%–0.23% of cases undergoing open-heart surgery [3]. SVAs most often arise from the right coronary sinus and are typically diagnosed after rupture [4]. With advances in imaging, however, unruptured cases are increasingly detected incidentally in asymptomatic patients. Although several studies have discussed the optimal timing of surgical intervention for unruptured SVAs, no clear guidelines or definitive criteria have yet been established [5, 6]. Therefore, the decision to intervene should be individualized based on clinical symptoms, associated complications, and anatomical features. In our case, surgery was indicated for a 25 mm aneurysm associated with severe AR.

There are various surgical options for the treatment of SVAs, including direct closure, patch repair, valve-sparing procedures, and aortic root replacement. In general, the surgical procedure is selected depending on the extent of the aortic root lesion and the condition of the coexisting valve. As Van Son et al. point out, although patch closure and valve repair often provide durable results in ruptured SVA, the presence of aortic root distortion and/or cusp lesions raises concerns about late recurrence of AR, suggesting that reintervention may be necessary [7]. In contrast, the Bentall procedure provides comprehensive replacement of the aortic root and is therefore associated with a lower risk of late AR and reintervention [8]. In the present case, the presence of aortic valve thickening further supports the appropriateness and usefulness of performing the Bentall procedure. Thus, the Bentall procedure represents a reliable option with low recurrence, particularly for unruptured SVAs treated electively. Our case supports this strategy, demonstrating that timely Bentall repair can achieve durable anatomical and functional outcomes in anatomically complex unruptured SVA.
